# Supplementary material for: Nutrition and Health in Arab Adolescents (NaHAR): Study protocol for the determination of ethnic-specific body fat and anthropometric cut-offs to identify metabolic syndrome
Source: PLoS One. 2024 Feb 23;19(2):e0298306. doi: 10.1371/journal.pone.0298306 (PMC10889849; doi:10.1371/journal.pone.0298306)
Supplement: Attachment — Submitted filename: v2 Reviewers comments.docx [file pone.0298306.s001.docx]

Journal Requirements:

When submitting your revision, we need you to address these additional requirements.

1. Please ensure that your manuscript meets PLOS ONE's style requirements, including those for file naming. The PLOS ONE style templates can be found at

<https://journals.plos.org/plosone/s/file?id=wjVg/PLOSOne_formatting_sample_main_body.pdf> and

[https://journals.plos.org/plosone/s/file?id=ba62/PLOSOne_formatting_sample_title_authors_affiliations.pdf](https://eur03.safelinks.protection.outlook.com/?url=https%3A%2F%2Fjournals.plos.org%2Fplosone%2Fs%2Ffile%3Fid%3Dba62%2FPLOSOne_formatting_sample_title_authors_affiliations.pdf&data=05%7C01%7Cln10%40aub.edu.lb%7C894cd37342174d0e808808db656776e6%7Cc7ba5b1a41b643e9a1206ff654ada137%7C1%7C1%7C638215267287422574%7CUnknown%7CTWFpbGZsb3d8eyJWIjoiMC4wLjAwMDAiLCJQIjoiV2luMzIiLCJBTiI6Ik1haWwiLCJXVCI6Mn0%3D%7C3000%7C%7C%7C&sdata=jOJmG0s6TrCP2Bbt5geG2yn%2BkG5b9H9pvbSvD5OnBxQ%3D&reserved=0)

Response: The manuscript has been reviewed and edited based on the PLOS ONE’s style templates provided by the editor.

2. We noticed you have some minor occurrence of overlapping text with the following previous publication(s), which needs to be addressed:

<https://www.liebertpub.com/doi/10.1089/met.2019.0033>

<https://www.cambridge.org/core/journals/british-journal-of-nutrition/article/erosion-of-the-mediterranean-diet-among-adolescents-evidence-from-an-eastern-mediterranean-country/569D34E53A9274EF4A37AE0DD6C81113>

In your revision ensure you cite all your sources (including your own works), and quote or rephrase any duplicated text outside the methods section. Further consideration is dependent on these concerns being addressed.

Response: Thank you for your comment. The sections have been revised. The above mentioned references were also cited.

3. We note that the grant information you provided in the ‘Funding Information’ and ‘Financial Disclosure’ sections do not match.

When you resubmit, please ensure that you provide the correct grant numbers for the awards you received for your study in the ‘Funding Information’ section.

Response: Sure, will do.

4. Thank you for stating the following financial disclosure:

“The study is funded by International Atomic Energy Agency (IAEA), and supported through collaboration among ARASIA State Parties under the framework of the IAEA technical cooperation project RAS6094, ‘Applying Nuclear Techniques for the Determination of Body Fat and Anthropometric Cutoffs (ARASIA)’. (ARASIA is a Cooperative Agreement for Arab States in Asia for Research, Development and Training related to Nuclear Science and Technology).

There are no grants awarded to individual researchers in the respective countries. Instead, each of the countries receive  the supplies and deuterium from the IAEA but there are no grants per se.”

Please state what role the funders took in the study.  If the funders had no role, please state: "The funders had no role in study design, data collection and analysis, decision to publish, or preparation of the manuscript." If this statement is not correct you must amend it as needed.

Response: The role of the funder has been clarified, as follows:

“This work was supported by the International Atomic Energy Agency (Technical Cooperation Programme; RAS6094). The IAEA provided technical support to the countries and participated in the design, preparation, review, and approval of the manuscript.”

Please include this amended Role of Funder statement in your cover letter; we will change the online submission form on your behalf.

Response: This has been included in the cover letter.

5. Please review your reference list to ensure that it is complete and correct. If you have cited papers that have been retracted, please include the rationale for doing so in the manuscript text, or remove these references and replace them with relevant current references. Any changes to the reference list should be mentioned in the rebuttal letter that accompanies your revised manuscript. If you need to cite a retracted article, indicate the article’s retracted status in the References list and also include a citation and full reference for the retraction notice.

Response: The reference list has been reviewed. We ensure that it is complete, correct and without any retracted articles.

[Note: HTML markup is below. Please do not edit.]

Reviewers' comments:

Reviewer's Responses to Questions

**Comments to the Author**

1. Does the manuscript provide a valid rationale for the proposed study, with clearly identified and justified research questions?

The research question outlined is expected to address a valid academic problem or topic and contribute to the base of knowledge in the field.

Reviewer #1: Yes

Reviewer #2: Yes

2. Is the protocol technically sound and planned in a manner that will lead to a meaningful outcome and allow testing the stated hypotheses?

The manuscript should describe the methods in sufficient detail to prevent undisclosed flexibility in the experimental procedure or analysis pipeline, including sufficient outcome-neutral conditions (e.g. necessary controls, absence of floor or ceiling effects) to test the proposed hypotheses and a statistical power analysis where applicable. As there may be aspects of the methodology and analysis which can only be refined once the work is undertaken, authors should outline potential assumptions and explicitly describe what aspects of the proposed analyses, if any, are exploratory.

Reviewer #1: Yes

Reviewer #2: Partly

3. Is the methodology feasible and described in sufficient detail to allow the work to be replicable?

Descriptions of methods and materials in the protocol should be reported in sufficient detail for another researcher to reproduce all experiments and analyses. The protocol should describe the appropriate controls, sample size calculations, and replication needed to ensure that the data are robust and reproducible.

Reviewer #1: Yes

Reviewer #2: Yes

4. Have the authors described where all data underlying the findings will be made available when the study is complete?

The [PLOS Data policy](https://eur03.safelinks.protection.outlook.com/?url=https%3A%2F%2Fjournals.plos.org%2Fplosone%2Fs%2Fmaterials-and-software-sharing&data=05%7C01%7Cln10%40aub.edu.lb%7C894cd37342174d0e808808db656776e6%7Cc7ba5b1a41b643e9a1206ff654ada137%7C1%7C1%7C638215267287422574%7CUnknown%7CTWFpbGZsb3d8eyJWIjoiMC4wLjAwMDAiLCJQIjoiV2luMzIiLCJBTiI6Ik1haWwiLCJXVCI6Mn0%3D%7C3000%7C%7C%7C&sdata=w%2FcgS6qFvJxkueSLuMQOhAcy3E4AlIxt4GULdVg72lU%3D&reserved=0) requires authors to make all data underlying the findings described in their manuscript fully available without restriction, with rare exception, at the time of publication. The data should be provided as part of the manuscript or its supporting information, or deposited to a public repository. For example, in addition to summary statistics, the data points behind means, medians and variance measures should be available. If there are restrictions on publicly sharing data—e.g. participant privacy or use of data from a third party—those must be specified.

Reviewer #1: No

Reviewer #2: No

Response: Data underlying the findings will be provided through a supporting file or a database link, when the study is complete.

5. Is the manuscript presented in an intelligible fashion and written in standard English?

PLOS ONE does not copyedit accepted manuscripts, so the language in submitted articles must be clear, correct, and unambiguous. Any typographical or grammatical errors should be corrected at revision, so please note any specific errors here.

Reviewer #1: Yes

Reviewer #2: Yes

6. Review Comments to the Author

Please use the space provided to explain your answers to the questions above and, if applicable, provide comments about issues authors must address before this protocol can be accepted for publication. You may also include additional comments for the author, including concerns about research or publication ethics.

You may also provide optional suggestions and comments to authors that they might find helpful in planning their study.

(Please upload your review as an attachment if it exceeds 20,000 characters)

Reviewer #1: Journal: PLOS ONE

Title: Nutrition and Health in Arab Adolescents (NaHAR): Study Protocol for the
Determination of Ethnic-Specific Body Fat and Anthropometric Cut-offs to Identify
Metabolic Syndrome

Reviewer’s comments

Sample size:
The manuscript used 210 and a minimum of 210 interchangeably for sample size. The authors should be clear about how many subjects will be included in each country.

Response: Thank you for pointing this to us. We have removed the term “minimum” as the sample size from each country is 210 adolescents. This has been clarified in the revised manuscript.

Under data collection:
How many schools will be included in each country needs to be made clear. If your sample size is 210 and all students in grades 10-12 are invited to participate, what do you do with those children whose parents have consented for them to participate after you reach your sample size? It is unclear how the sample size will be maintained unless you have a pre-determined number of schools selected for each country and the number of students to participate in the study per school. Extra care should also be taken not to overburden parents when you know that only a few students will be selected for the study in a particular school.

Response: We thank the reviewer for this comment. We have clarified in our revised manuscript that 4-5 schools will be approached in each country, whereby 45-50 students will be recruited from each school. This has been stated in the manuscript as follows: “In each country, a sample of 210 adolescents aged 15-18 years will be recruited from 4-5 local schools.” (Lines 142-143).

As for the consent process and based on previous studies, refusal rate is usually high in such studies involving adolescents. We will make sure to keep a daily log on the number of participants we have recruited before inviting others to join.

Under Anthropometrics:
Provide the company(manufacturer) names and locations for each tool used. E.g., Seca scale, measuring tape for WC and MUAC, D2O for DDL, and Fourier Transform Infrared (FTIR) spectrometry. Provide details similar to what you did for the BIVA device (BIA 101, Akern, Via Lisbona, Italy)
WHO Anthroplus software(provide a citation)

Response: The company names and locations have been added for the seca scale, stadiometer, measuring tape for WC, MUAC and calf circumference, as well as the D2O and FTIR. Moreover, we provided the following citation for the Anthroplus software:

World Health Organization. Growth reference data for 5-19 years. Application tools: WHO AnthroPlus software. [cited 2023 July 4]. Available from: <https://www.who.int/tools/growth-reference-data-for-5to19-years/application-tools>.

Ethical considerations:
“Country-specific data will be pooled within a regional database for data analysis.” it is unclear why this statement is here.

Response: We thank you for pointing this to us. The statement has been removed from the manuscript.

Statistical analysis:
Lines#309,310: Chi-square and independent t-tests. Specify which test is for categorical and which is for continuous variables.

Response: Based on the reviewer’s comment, we have revised this section, as follows:

“A comparison between participants with and without MetS will be conducted using Chi-square for categorical variables and independent t-tests for continuous variables”. (Lines 321-323)

Lines#317,318 and 322-324. What is the purpose of performing repeated ANOVA and paired t-tests on DDL vs. BIVA? What additional information will both tests provide instead of just one?

Response: We thank you for pointing this to us. There is in fact no need for ANOVA. We have edited this section, as follows: “Differences in %BF estimates derived from DDL and BIVA will be assessed using paired t-test”. (Line 330)

In fact, this analysis will help us in addressing our secondary objective related to examining the validity of Bioelectrical Impendence Vector Analysis (BIVA) in estimating BF against the deuterium dilution technique (DDL).


Line# 335. Use sentence case formatting as done for the other headings.

Response: Done.

Reviewer #2: The manuscript entitled "Nutrition and Health in Arab Adolescents ( 1 NaHAR): Study Protocol for the Determination of Ethnic-Specific Body Fat and Anthropometric Cut-offs to Identify Metabolic Syndrome” is led by Nasreddine L et al., from a multicenter and multinational group of investigators. The strong team is set to achieve the goals listed in the protocol review. The study will contribute to the validation of anthropometric cut-off points in the middle-east population of adolescents with excess adiposity and associated metabolic syndrome risks.

The following points are written to improve their study:

1. Investigators from Jordan is still not have approved ethical clearance. Authors should clearly state whether it is okay to have this protocol publication before the approval of investigation in one site.

Response: Jordan has obtained ethical clearance. The statement has been edited as follows: “Ethical approval for the study was already obtained from the Institutional Review Board of the American University of Beirut (BIO-2021-0022) in Lebanon, the Institutional Review Board of King Abdullah Bin Abdulaziz University Hospital (Log number 22-0029) in KSA, the Institutional Committee of Bioethics in Syria (date September 1, 2021), the Ministry of Health for Planning and Quality Affairs (Research number 2134/2022) in Kuwait, and the Ministry of Health (date June 3, 2021 – Number 23221 /8/1/2) in Jordan.” (Lines 306-311)

2. How was the sample size calculated? The sample size of 210 is sufficient for their population, without statistical analysis for sample size, this becomes a weakness.

Response: The sample size was calculated using the STEP sample size calculator. This has been clarified in the manuscript as follows: “The sample size of 210 subjects was calculated using the STEPS Sample Size Calculator and Sampling Spreadsheet, using a margin of error of 5%, a confidence interval (CI) of 95% and a prevalence of MetS among adolescents of 10%.” (Lines 148-151)

Reference: World Health Organization. STEPS Sample Size Calculator and Sampling Spreadsheet. [cited 2020 Nov 9]. Available from: <https://cdn.who.int/media/docs/default-source/ncds/ncd-surveillance/steps/sample-size-calculator.xls?sfvrsn=ee1f4ae8_2>.

3. Authors should consider food frequency questionnaire (FFQ) to monitor dietary intake

Response: We thank you for this suggestion. Unfortunately, we do not have a validated FFQ in each of the countries where data collection is being conducted.

4. Authors can also consider measuring liver function test in the blood, as part of metabolic syndrome and this data will be useful in addition not lipid biomarkers in the adolescent population.

Response: Indeed this would provide valuable insight as liver enzymes may be affected by obesity and metabolic abnormalities. However, we have opted to adhere to the official definition of the metabolic syndrome. For consistency purposes, we have also removed glycated Hb measurements from the manuscript, so we are fully in line with the official definition of the MetS.

We have also addressed this point in the limitations section, as follows:

“In addition, the identification of metabolic abnormalities in this study is restricted to those included in the IDF definition of the MetS and thus other parameters that may be influenced by obesity such as liver function tests or inflammatory markers are excluded.”

7. PLOS authors have the option to publish the peer review history of their article ([what does this mean?](https://eur03.safelinks.protection.outlook.com/?url=https%3A%2F%2Fjournals.plos.org%2Fplosone%2Fs%2Feditorial-and-peer-review-process%23loc-peer-review-history&data=05%7C01%7Cln10%40aub.edu.lb%7C894cd37342174d0e808808db656776e6%7Cc7ba5b1a41b643e9a1206ff654ada137%7C1%7C1%7C638215267287422574%7CUnknown%7CTWFpbGZsb3d8eyJWIjoiMC4wLjAwMDAiLCJQIjoiV2luMzIiLCJBTiI6Ik1haWwiLCJXVCI6Mn0%3D%7C3000%7C%7C%7C&sdata=zsA4W85CKzch91IeVP6BROsnKfW6%2B%2Bc3YAJhJ45EqVU%3D&reserved=0)). If published, this will include your full peer review and any attached files.

If you choose “no”, your identity will remain anonymous but your review may still be made public.

**Do you want your identity to be public for this peer review?** For information about this choice, including consent withdrawal, please see our [Privacy Policy](https://eur03.safelinks.protection.outlook.com/?url=https%3A%2F%2Fwww.plos.org%2Fprivacy-policy&data=05%7C01%7Cln10%40aub.edu.lb%7C894cd37342174d0e808808db656776e6%7Cc7ba5b1a41b643e9a1206ff654ada137%7C1%7C1%7C638215267287578818%7CUnknown%7CTWFpbGZsb3d8eyJWIjoiMC4wLjAwMDAiLCJQIjoiV2luMzIiLCJBTiI6Ik1haWwiLCJXVCI6Mn0%3D%7C3000%7C%7C%7C&sdata=6vz%2FPMFXk0sqHKBPZGaCx7mylWfceJ5VAOsdz0sSMGY%3D&reserved=0).

Reviewer #1: No

Reviewer #2: No
