# Supplementary material for: Nutrition and Health in Arab Adolescents (NaHAR): Study protocol for the determination of ethnic-specific body fat and anthropometric cut-offs to identify metabolic syndrome
Source: PLoS One. 2024 Feb 23;19(2):e0298306. doi: 10.1371/journal.pone.0298306 (PMC10889849; doi:10.1371/journal.pone.0298306)
Supplement: Attachment — Submitted filename: Response to Reviewers.docx [file pone.0298306.s002.docx]

**October 16, 2023**

**Nutrition and Health in Arab Adolescents (NaHAR): Study Protocol for the Determination of Ethnic-Specific Body Fat and Anthropometric Cut-offs to Identify Metabolic Syndrome**

**Manuscript ID:** PONE-D-23-02201R1

We would like to thank the editor and the reviewers for the constructive comments on our manuscript. Please find below a point by point response to each of the comments provided by the reviewers.

**Journal Requirements:**

Please review your reference list to ensure that it is complete and correct. If you have cited papers that have been retracted, please include the rationale for doing so in the manuscript text, or remove these references and replace them with relevant current references. Any changes to the reference list should be mentioned in the rebuttal letter that accompanies your revised manuscript. If you need to cite a retracted article, indicate the article’s retracted status in the References list and also include a citation and full reference for the retraction notice.

Response to comment: The reference list has been reviewed to ensure that it is complete, correct and without any retracted articles.
 **Additional Editor Comments:**

The authors should address the minor comment given by the reviewer


[Note: HTML markup is below. Please do not edit.]

Reviewers' comments:

Reviewer's Responses to Questions

**Comments to the Author**

1. Does the manuscript provide a valid rationale for the proposed study, with clearly identified and justified research questions?

The research question outlined is expected to address a valid academic problem or topic and contribute to the base of knowledge in the field.

Reviewer #2: Yes

Reviewer #3: Yes

2. Is the protocol technically sound and planned in a manner that will lead to a meaningful outcome and allow testing the stated hypotheses?

The manuscript should describe the methods in sufficient detail to prevent undisclosed flexibility in the experimental procedure or analysis pipeline, including sufficient outcome-neutral conditions (e.g. necessary controls, absence of floor or ceiling effects) to test the proposed hypotheses and a statistical power analysis where applicable. As there may be aspects of the methodology and analysis which can only be refined once the work is undertaken, authors should outline potential assumptions and explicitly describe what aspects of the proposed analyses, if any, are exploratory.

Reviewer #2: Yes

Reviewer #3: Yes

3. Is the methodology feasible and described in sufficient detail to allow the work to be replicable?


Descriptions of methods and materials in the protocol should be reported in sufficient detail for another researcher to reproduce all experiments and analyses. The protocol should describe the appropriate controls, sample size calculations, and replication needed to ensure that the data are robust and reproducible.

Reviewer #2: Yes

Reviewer #3: Yes

4. Have the authors described where all data underlying the findings will be made available when the study is complete?

The [PLOS Data policy](https://journals.plos.org/plosone/s/materials-and-software-sharing) requires authors to make all data underlying the findings described in their manuscript fully available without restriction, with rare exception, at the time of publication. The data should be provided as part of the manuscript or its supporting information, or deposited to a public repository. For example, in addition to summary statistics, the data points behind means, medians and variance measures should be available. If there are restrictions on publicly sharing data—e.g. participant privacy or use of data from a third party—those must be specified.

Reviewer #2: No

Reviewer #3: Yes

Response to comment: We have added the following statement to our protocol paper in order to clarify where the data underlying the findings can be found:

“Data underlying the findings will be provided through a supporting file or a database link, when the study is complete.” (Lines 423-424)

5. Is the manuscript presented in an intelligible fashion and written in standard English?

PLOS ONE does not copyedit accepted manuscripts, so the language in submitted articles must be clear, correct, and unambiguous. Any typographical or grammatical errors should be corrected at revision, so please note any specific errors here.

Reviewer #2: Yes

Reviewer #3: Yes

6. Review Comments to the Author

Please use the space provided to explain your answers to the questions above and, if applicable, provide comments about issues authors must address before this protocol can be accepted for publication. You may also include additional comments for the author, including concerns about research or publication ethics.

You may also provide optional suggestions and comments to authors that they might find helpful in planning their study.


(Please upload your review as an attachment if it exceeds 20,000 characters)

**Reviewer #2:**

Authors have improved their protocol. I would still suggest them to include Harvard Willet FFQ for their study and liver function test would be additional work, but would provide great knowledge. However, this is a protocol and their choice is okay

Response to comment: We thank the reviewer for the valuable comments on our manuscript. We agree that a food frequency questionnaire would provide important data on the habitual intake of participating subjects. Although the Harvard Willet FFQ has been shown to be a valid assessment tool in the USA (and other countries with similar dietary practices), its validity has not been ascertained in countries that may have significantly different dietary habits (such as countries of the region). There is an urgent need to develop and validate a FFQ that is specific to Arab countries. We have acknowledged this point in our limitations section, as follows:

“Moreover, in the absence of a validated food frequency questionnaire for countries in the region, dietary assessment is conducted based on a 24 hour dietary recall.” (Lines 397-399)

We do also agree with the added value of liver tests, however it will be difficult for us to implement it because of budgetary implications as well as for consistency with the official definition of the metabolic syndrome. We have acknowledged this in the limitation section as follows:

“In addition, the identification of metabolic abnormalities in this study is restricted to those included in the IDF definition of the MetS and thus other parameters that may be influenced by obesity such as liver function tests or inflammatory markers are excluded.” (Lines 394-397)

**Reviewer #3:**

INTRODUCTION

Lines 112 and 113
•Why did the authors not put under consideration other core elements for anthropometry such as waist-to-hip ratio, thigh-to-hip ratio, and skinfold thickness? This has also been used in measuring adiposity.

Response to comment: We thank the reviewer for this comment. Indeed these measurements/ratios (waist to hip, thigh to hip; skinfold thicknesses) have been previously used in assessing adiposity. However, in our study, we tried to focus on the most common, and field-appropriate anthropometric indicators including BMI, WC, WHtR and MUAC. These indicators are practical, inexpensive and relatively easy to use in large field surveys.

Although useful in certain contexts, skinfold thicknesses are difficult to measure with precision and accuracy without rigorous training, and hence may not be very suited to field-based assessments. These measurements have been also described to be subject to considerable inter- and intra-observer error in the measurements. It may be difficult to pick up a consistent fold of skin and subcutaneous fat and, without proper care and attention, the use of the calipers may causes pain or bruising. In the very obese, the skinfold may also sometimes be larger than the calipers can measure.

References:

Wendel, D., Weber, D., Leonard, M. B., Magge, S. N., Kelly, A., Stallings, V. A., ... & Zemel, B. S. (2017). Body composition estimation using skinfolds in children with and without health conditions affecting growth and body composition. *Annals of human biology*, *44*(2), 108-120.

Eaton–Evans, J. (2005). Nutritional assessment| Anthropometry. <https://www.sciencedirect.com/science/article/pii/B9780123750839001975?via%3Dihub>

The waist to hip ratio may also provide valuable information on body fat distribution, but it will necessitate obtaining additional measurements of the hip circumferences which may decrease its practical acceptability or feasibility. In addition, various studies have suggested that waist circumference or waist to height ratio are better correlated with body fat and/or cardiovascular risk than the waist to hip ratio.

References:

Ashtary-Larky, D., Daneghian, S., Alipour, M., Rafiei, H., Ghanavati, M., Mohammadpour, R., ... & Afrisham, R. (2018). Waist circumference to height ratio: better correlation with fat mass than other anthropometric indices during dietary weight loss in different rates. *International journal of endocrinology and metabolism*, *16*(4).

Bacopoulou, F., Efthymiou, V., Landis, G., Rentoumis, A., & Chrousos, G. P. (2015). Waist circumference, waist-to-hip ratio and waist-to-height ratio reference percentiles for abdominal obesity among Greek adolescents. *BMC pediatrics*, *15*(1), 1-9.

Gažarová, M., Bihari, M., Lorková, M., Lenártová, P., & Habánová, M. (2022). The Use of different anthropometric indices to assess the body composition of Young Women in Relation to the incidence of obesity, Sarcopenia and the premature mortality risk. *International Journal of Environmental Research and Public Health*, *19*(19), 12449.

Caminha, T. C., Ferreira, H. S., Costa, N. S., Nakano, R. P., Carvalho, R. E. S., Xavier Jr, A. F., & Assunção, M. L. (2017). Waist-to-height ratio is the best anthropometric predictor of hypertension: a population-based study with women from a state of northeast of Brazil. *Medicine*, *96*(2).

We agree that there is potential for thigh circumference or the thigh-to-hip ratio to be a proxy for adiposity (or LBM). However, the use of these indicators may be further complicated by its cultural acceptability and feasibility.

Reference:

McCarthy, H. D. (2014). Conference on ‘Childhood nutrition and obesity: current status and future challenges’ Symposium 2: Data collection; Measuring growth and obesity across childhood and adolescenc. *PROCEEDINGS OF THE NUTRITION SOCIETY*, *73*, 210-217.

We have acknowledged this point in the limitations section as follows: “Finally, this study does not comprise a comprehensive set of anthropometric indicators but rather focuses on the most common and field-appropriate ones, including BMI, WC, WHtR and MUAC, as these were shown to be practical, inexpensive and relatively easy to use in large field surveys.” (Lines 399-402).

Line 121- 124.
•I would advise revising the sentence to be more inclusive of the study objectives, not only anthropometrics cut-offs. i.e. will the study not bring validated Body Fats measurements?

Response to comment: as recommended by the reviewer, this sentence has been revised to the following: “This study will therefore contribute to the generation of validated anthropometric and body fat cut-off points”.

MATERIALS AND METHODS
Table 1. What types of illnesses among the wide range of inborn metabolic disorders do you specifically mean when you say "those with inborn errors of metabolism"? and How will they be recognized for the purpose of exclusion.

Response to comment: We thank the reviewer for this comment. We have clarified in our revised manuscript that this exclusion will be based on whether the subject reports a previous diagnosis of inborn errors of intermediary metabolism (that are mainly related amino acids, carbohydrates, and fatty acids’ metabolism). This will be ascertained via a screening questionnaire that will be administered to the subjects to assess their eligibility to participate in the study.

To address the reviewer’s comment, Table 1 was modified as well as lines 171-175 where we clarified the following: “Following a specific protocol, eligibility of adolescents who agreed to be part of the study, and whose parents/legal guardians have consented, will be confirmed based on a screening questionnaire that inquires about age, nationality, health/medication status, puberty stage, measured weight and height (and body mass index consequently”.

Deuterium Dilution Technique:
•Due to the circadian effect on saliva. It is recommended to collect saliva at a specific time. Does this apply to this study? Authors should revise thoroughly on methods for saliva collection. Including the exclusion of participants with any oral illness like xerostomia and others that may affect the saliva flow rate and therefore distort the results. For more insights about saliva collection methods authors can read this paper <https://doi.org/10.3390/oral3030027>

Response to comment: We thank you for your comment.

As suggested by the reviewer, we have amended the exclusion criteria to exclude those reporting to have oral illnesses like xerostomia and others that may affect the saliva flow. This is shown in Table 1 in the revised manuscript.

We agree that there may well be circadian variation in saliva production and in saliva composition, but not in relation to the behaviour of the isotope in the period between dosing and post dose sampling (Coward 1982). Since Deuterium is in equilibrium with Hydrogen throughout the body water after 3-4 hours, saliva production or composition is not considered to have an influence on Deuterium enrichment (Coward 1982)..

Reference: Coward, W. A., Cole, T. J., Sawyer, M. B., & Prentice, A. M. (1982). Breast-milk intake measurement in mixed-fed infants by administration of deuterium oxide to their mothers. *Human nutrition. Clinical nutrition*, *36*(2), 141-148.

•Will participants be allowed to eat or drink anything before post-dose saliva collection? •If “Yes” will it not bring errors in Salimetrics
•If "No," authors should explain how they will restrict eating since participants must fast for at least eight hours in
order to measure blood parameters that need fasting and as a prerequisite for saliva collection. Three more hours would jeopardize the participants and some may hesitate to participate

Response to comment: Thank you for this comment. Yes, we have clarified this point in the revised manuscript as follows: “As per the IAEA recommended procedure for deuterium dilution, all participants are given a standardized dry snack and a juice of approximately 200 ml about one hour after dose administration, and at least 30 minutes before the post-dose saliva sampling.” (Lines 225-228).

In fact this IAEA procedural guidance document has explained that “To allow full and fast absorption of the dose, it is recommended that the participant does not drink or eat anything until 30 minutes after dosing. In addition, to avoid dilution of the deuterium enrichment in saliva, no food or drink should be taken during the last 30 minutes before the post-dose saliva samples are collected. Consider giving a standardized snack and a drink (preferably water) of up to 250 mL to children and adults 1 to 2 hours after dosing.”.

Status and Timeline of the study
The table is not clear. Have the authors begun the data collection?

Response to comment: The table has been amended as the timeline of the project has changed since our first submission to the journal. We have also provided additional clarifications as a legend to the table.

Recruitment and data collection (mainly pilot-testing) was initiated in some sites. Sample analysis pertinent to blood and saliva is planned to be initiated in parallel to data collection (in batches), but it is also expected to continue for a couple of months after data collection ends (i.e. beginning of year 2025). Data analysis and reporting will be performed during 2024 and beginning of 2025.

7. PLOS authors have the option to publish the peer review history of their article ([what does this mean?](https://journals.plos.org/plosone/s/editorial-and-peer-review-process#loc-peer-review-history)). If published, this will include your full peer review and any attached files.


If you choose “no”, your identity will remain anonymous but your review may still be made public.

**Do you want your identity to be public for this peer review?** For information about this choice, including consent withdrawal, please see our [Privacy Policy](https://www.plos.org/privacy-policy).

Reviewer #2: No

Reviewer #3: **Yes:** Oscar Mbembela
